# TMalphaDB and TMbetaDB: web servers to study the structural role of sequence motifs in α-helix and β-barrel domains of membrane proteins

**DOI:** 10.1186/s12859-015-0699-5

**Published:** 2015-08-20

**Authors:** Marc Perea, Ivar Lugtenburg, Eduardo Mayol, Arnau Cordomí, Xavier Deupí, Leonardo Pardo, Mireia Olivella

**Affiliations:** Laboratori de Medicina Computacional, Unitat de Bioestadística, Facultat de Medicina, Universitat Autònoma de Barcelona, Bellaterra, Spain; Department de Biologia de Sistemes, Universitat de Vic, Vic, Barcelona, Spain; Present address: Condensed Matter Theory Group and Laboratory of Biomolecular Research, Paul Scherrer Institut, Villigen PSI, Switzeland

**Keywords:** Membrane proteins, Transmembrane segments, Sequence motifs, Structural distortion

## Abstract

**Background:**

Membrane proteins represent over 25 % of human protein genes and account for more than 60 % of drug targets due to their accessibility from the extracellular environment. The increasing number of available crystal structures of these proteins in the Protein Data Bank permits an initial estimation of their structural properties.

**Description:**

We have developed two web servers—TMalphaDB for α-helix bundles and TMbetaDB for β-barrels—to analyse the growing repertoire of available crystal structures of membrane proteins. TMalphaDB and TMbetaDB permit to search for these specific sequence motifs in a non-redundant structure database of transmembrane segments and quantify structural parameters such as ϕ and ψ backbone dihedral angles, χ_1_ side chain torsion angle, unit bend and unit twist.

**Conclusions:**

The structural information offered by TMalphaDB and TMbetaDB permits to quantify structural distortions induced by specific sequence motifs, and to elucidate their role in the 3D structure. This specific structural information has direct implications in homology modeling of the growing sequences of membrane proteins lacking experimental structure. TMalphaDB and TMbetaDB are freely available at http://lmc.uab.cat/TMalphaDB and http://lmc.uab.cat/TMbetaDB.

## Background

Membrane proteins represent over 25 % of all proteins in sequenced genomes and mediate the interaction of the cell with its surroundings, including selective molecular transport, signalling, respiration and motility [1]. Because of their accessibility from the extracellular environment, membrane proteins are targets of over 60 % of currently marketed drugs [2–4]. Due to the difficulty in over-expressing, purifying and crystallizing membrane proteins [5], only 2 % of the structures deposited in Protein Data Bank are membrane proteins [6, 7]. Membrane proteins display specific features that differ from those of water-soluble ones, due to their different environment [8]. For instance, the number of folds that membrane proteins can adopt is limited to α-helix bundles and β-barrels due to the physical constraints imposed by the lipid bilayer. The lipid bilayer, where the transmembrane (TM) regions are located, is predominantly lipophilic, lacks hydrogen-bonding potential, and provides little screening of electrostatic interactions. Thus, α-helix and β-sheets secondary structure elements maximize the hydrogen bond interactions among backbone atoms, whereas hydrophobic side chains are preferentially oriented toward the membrane lipids. This results in significant differences in amino acid composition [9] and in the probabilities of amino acid substitutions during evolution [10, 11] relative to globular proteins.

Biological function of membrane proteins involves conformational rearrangement of the TM regions. For example, activation of the G protein-coupled receptor family requires the binding of the C-terminal α-helix of the G protein to the intracellular cavity that is opened by the conformational rearrangement of TM6 [12]. Similarly, multidrug transporters are flexible proteins that switch from outward-open to inward-open conformations, facilitating the release of the substrate [13]. Such conformational changes require local flexibility or distortions in the TM regions, which can be provided by specific structural motifs. For instance, our laboratory has shown that serine or threonine, either alone [14] or in combination with proline [15], induces distinctive TM distortions to accommodate the structural needs of specific protein functions [16, 17]. To address this issue we have developed two non-redundant databases of 3D structures of TM segments consisting on α-helix bundles and β-barrels that are accessible through the TMalphaDB and TMbetaDB web servers, respectively. The main advantage of these servers is their ability to systematically survey sequences of TM regions and provide to the users main structural parameters, such as backbone ϕ and ψ dihedral angles and side chain χ_1_ angle, as well as helix bend and twist angles. This structural information allows to quantify distortions induced by residues or motifs and to elucidate their role in the structure and function of membrane proteins.

## Construction and content

TMalphaDB and TMbetaDB are web-based servers that combine a MySQL database management system and Python programs with a dynamic web interface based on PHP.

### Non-redundant databases of transmembrane segments structures of alpha and beta membrane proteins

TMalphaDB and TMbetaDB currently contain 330 structures of α-helix bundles and 107 structures of β-barrels, respectively, with a resolution lower than 3.5 Å. To avoid redundancy, only one structure for each protein is selected (i.e. one structure per UniProt accession code). Among different structures with the same UniProt accession code, the one with best resolution and resemblance to the native state (i.e. without mutations, native pH) is selected. Additionally, for multimeric proteins, only one subunit is extracted. The complete list of structures, together with the unique subunit database, can be downloaded at http://lmc.uab.cat/TMalphaDB/info.php and http://lmc.uab.cat/TMbetaDB/info.php. These databases are regularly and automatically updated, in order to include new solved proteins. Each structure is characterized by the Protein Data Bank identification code (PDBID) [18], protein name, Uniprot accession code [19], family name according to Orientations of Proteins in Membranes [20] and organism. Moreover, because the hydrophobic nature of the lipid bilayer conditions the structure and features of the membrane-embedded regions relative to the water-exposed ones [8, 11], we used PDBTM [7, 21] to download only the coordinates of the domain of the protein that is inserted in the lipid bilayer.

### Tools to analyse sequence and structure of membrane proteins

The importance of TMalphaDB and TMbetaDB is their ability to search and analyse specific residues or sequence motifs in TM segments of membrane proteins. The search is performed using the single-letter amino acid code or/and in combination with wildcard characters such as ‘+’ (positively charged, K/R), ‘−’ (negatively charged, D/E), ‘*’ (charged, K/R/H/D/E), ‘@’ (aromatic, F/W/Y/H), ‘~’ (hydrophobic, I/L/V/M/F/A/P), ‘^’ (polar, D/E/N/Q/K/R/H/S/T/C/W/Y), ‘%’ (aliphatic, L/V/I/M), ‘#’ (distorting, P/G), ‘?’ (hydroxylic, S/T/Y), ‘$’ (sulphur-containing, C/M), ‘.’ (tiny, G/A), ‘!’ (aromatic amphipathic, W/Y/H), or ‘x’ (any amino acid). Moreover, the search (advanced options) can be filtered by the proximity of the sequence motif to the beginning/end of the TM domain (as the structural parameters can be highly influenced by the loops) or the presence of certain amino acids within the sequence motif (as, for instance, Pro or/and Gly can distort the secondary structure conformation). The output consists of a list of proteins, identified by the PDBID, Uniprot accession code, the name and identifier of the first residue in the motif, the sequence of the TM segment with the requested motif highlighted and the family name of the protein. The coordinates of the entire protein, the TM segments and/or a unique subunit of the protein can be downloaded for each entry. The user can select all TM segments, unique TM segments (i.e. only one TM segments is select for repeated subunits), or a manually selection can be performed. Average backbone ϕ and ψ angles and side chain χ_1_ angle for the selected TM segments can be downloaded or/and displayed in a plot. When all the analysed sequences feature the same type of residue (according to the wildcards previously defined), for a specific position, the plot uses this representation. In TMalphaDB, bend and twist angles, two relevant parameters to measure local distortions of TM helices, are also calculated and plotted for the identified/selected TM segments using HELANAL [22]. Local bend angles are calculated as the angle between the axes of the cylinders formed by the Cα atoms of the residues preceding (*i-3*, *i*) and following (*i*, *i + 3*) a given amino acid *i*. Unit twist angles are calculated for sets of four consecutive Cα atoms, i.e. one turn, to analyze helical uniformity. An ideal α-helix, with approximately 3.6 residues per turn, has a unit twist of approximately 100° (360°/3.6). A closed helical segment, with <3.6 residues per turn, possesses a unit twist >100°, whereas an open helical segment, with >3.6 residues per turn, possesses an unit twist <100°. A variation greater than 20° in the unit twist angle will result in a change in the orientation of the amino acid side chain. Finally, JSMoL [23] sessions containing the coordinates of the requested motif, and all residues and ligands in its environment can also be displayed.

## Utility and discussion

Membrane proteins incorporate in the sequence of their TMs specific residues like Pro and Gly, introducing a flexible point and assisting in helix movements or stabilizing local regions of structural relevance [24]. In order to illustrate the use of TMalphaDB and TMbetaDB, we have surveyed and quantified structural distortions induced by P and PP motifs in TM α-helices and P and G residues in TM β-strands.

### P and PP motifs in TM α-helices

**Fig. 1:**
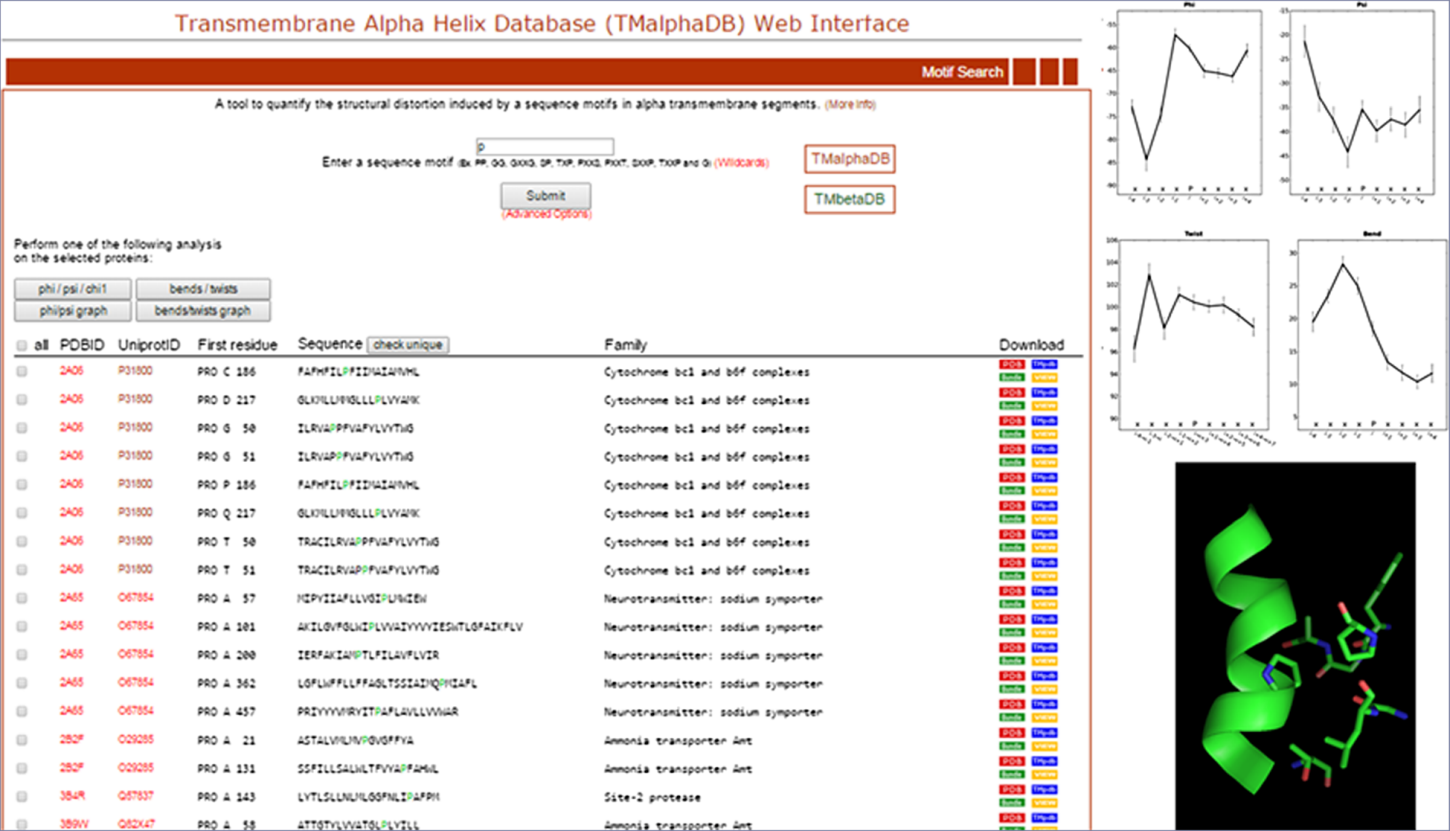
Snapshots of the TMalphaDB output. The output consists in a list of proteins containing the “P” motif (*left panel*), average backbone ϕ and ψ angles (*top right panel*), average bend and twist angles (*central right panel*), and a JSMol session displaying all residues and ligands at a distance cutoff of 5 Å from the “P” motif (*bottom right panel*)

**Fig. 2:**
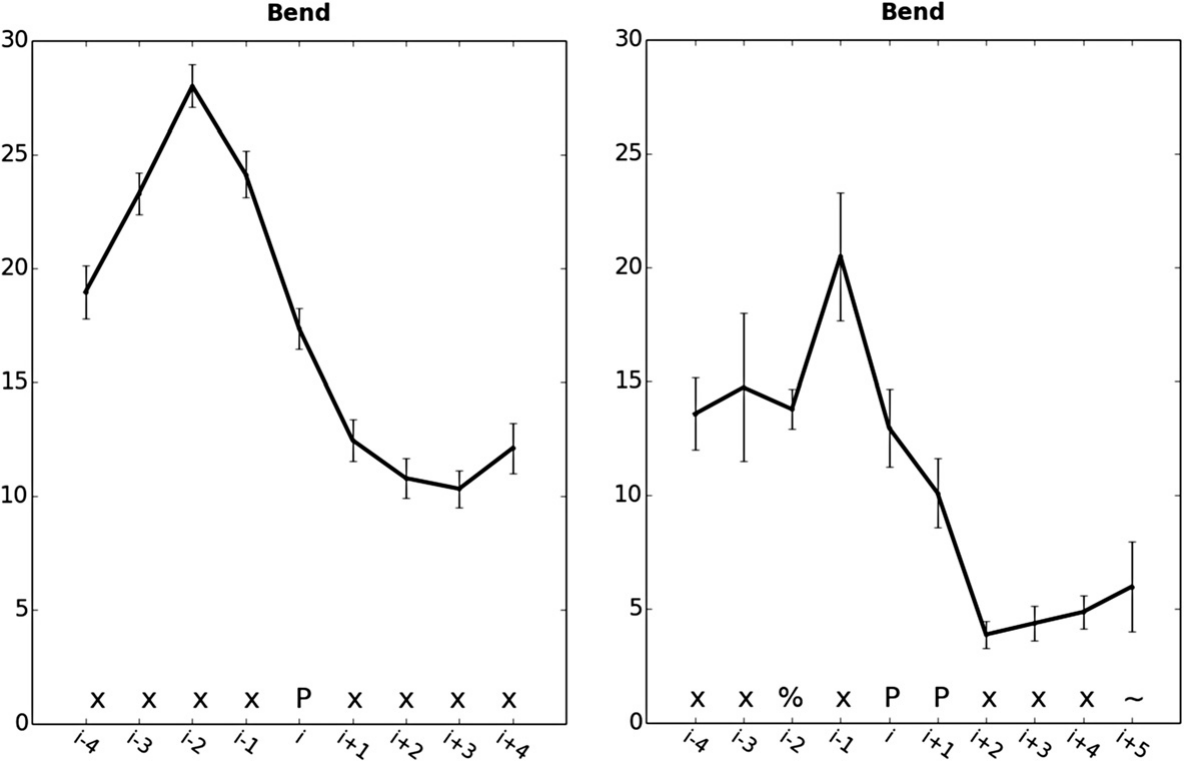
Bend angle of TM α-helices. Average bend angle of TM helices containing P (left panel;*n* = 349, “P” search) and PP (right panel; *n* = 8, “PP” search). Motifs located 4 positions from either the beginning/end of the TM domain or/and containing Pro/Gly within 4 residues of the motif were excluded

Although Pro presents the smallest helix-forming tendency among naturally occurring amino acids [25], Pro residues are often observed in TM helices [26] where they induce a significant distortion. This is produced to avoid a steric clash between the pyrrolidine ring of Pro and the carbonyl oxygen of the residue in the preceding turn, leading to a bending of the helical structure [27]. Moreover, two consecutive Pro residues are also observed in sequences of membrane proteins. In order to study the distortion induced by the PP motif, relative to P, we scanned TMalphaDB. The search resulted in 349 unique TM helices containing P (search=“P”) and 8 TM helices containing PP (search=“PP”). Figure 1 shows a snapshot of the obtained output for the “P” search, plots for phi and psi dihedrals and unit twist/bend and a Pymol session showing the residues located near “P”. The obtained average bend angle plots for P and PP are shown to compare the structural distortion induced by each sequence motif (Fig. 2a). Clearly, the distortion in bend induced by PP is lower than for P and is not the sum of individual Pro distortions, suggesting a modulating effect.

### P and G in TM β-barrels

**Fig. 3:**
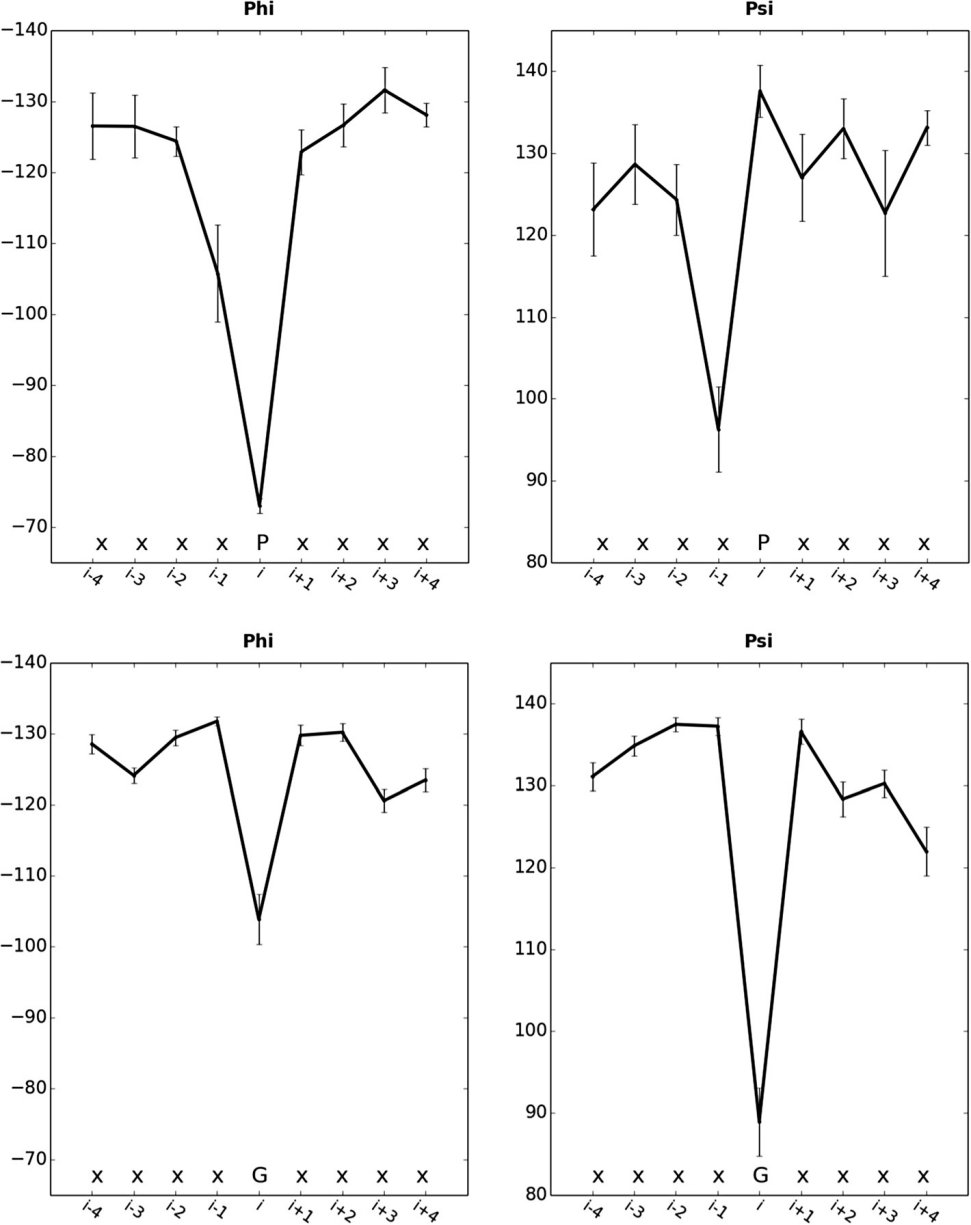
ϕ and ψ dihedral angles of TM β-strands. Average ϕ and ψ dihedral angles in TM β-strands containing P (*n* = 172 search=“P”) and G (*n* = 1031, search=“G”)

The cyclic structure of the side chain of Pro locks the ϕ dihedral angle at approximately -60°, which is incompatible with ϕ values near −130° observed in β-strands. We scanned TMbetaDB in order to calculate the backbone ϕ and ψ dihedral angles of Pro when located in β-barrel domains of membrane proteins. The TMbetaDB search resulted in 172 TM segments containing P whose average ϕ and ψ dihedral angles are plotted in Fig. 3. Relative to the energetically preferred ϕ and ψ dihedral angles near −130° and 130° of β-strands, Pro increases ϕ and triggers a decrease in ψ at position *i-1*. In contrast to Pro, the absence of a side chain in Gly allows high flexibility in the polypeptide chain as well as dihedral angles. In order to calculate the observed ϕ and ψ dihedral angles of Gly in β-barrel domains TMbetaDB was scanned. The search resulted in 1031 TM segments containing Gly. Figure 3 shows that, on average, Gly increases ϕ and decreases ψ dihedral angles. These results indicate that both Pro and Gly induce a distortion in the conformation of main polypeptide chain in TM β-strands.

## Conclusions

The structural data provided by TMalphaDB and TMbetaDB quantify structural distortions induced by specific amino acids or motifs, and elucidate their role in the structure of membrane proteins. This specific structural information can be, for instance, incorporated in the homology modelling of membrane proteins lacking experimental structure. Thus, these servers emerge as valuable tools to fill the growing gap between the pool of known sequences of membrane proteins and the number of experimentally determined structures.

## Availability and requirements

TMalphaDB and TMbetaDB are freely available at http://lmc.uab.cat/TMalphaDB and http://lmc.uab.cat/TMbetaDB.

## Abbreviation

TM: Transmembrane segments
PDBID: Protein Data Bank identification code
